# Mentorship and pursuit of academic medicine careers: a mixed methods study of residents from diverse backgrounds

**DOI:** 10.1186/1472-6920-14-26

**Published:** 2014-02-09

**Authors:** Baligh R Yehia, Peter F Cronholm, Nicholas Wilson, Steven C Palmer, Stephen D Sisson, Conair E Guilliames, Norma I Poll-Hunter, John-Paul Sánchez

**Affiliations:** 1 Department of Medicine, University of Pennsylvania Perelman School of Medicine, Philadelphia, Pennsylvania; 2 Department of Family Medicine and Community Health, University of Pennsylvania Perelman School of Medicine, Philadelphia, Pennsylvania; 3 Department of Psychiatry, University of Pennsylvania Perelman School of Medicine, Philadelphia, Pennsylvania; 4 Department of Medicine, Johns Hopkins University School of Medicine, Baltimore, Maryland; 5 Department of Family Medicine, Albert Einstein College of Medicine, Bronx, New York; 6 Diversity Policy and Programs, Association of American Medical Colleges, Washington, DC, USA; 7 Department of Emergency Medicine, Albert Einstein College of Medicine, Bronx, New York, USA; 8 1309 Blockley Hall, 423 Guardian Drive, Philadelphia 19104, Pennsylvania

**Keywords:** Residents, Postgraduate, Academic careers, Career choice, Diversity, Minority, Mentorship, Mentoring, Medical education

## Abstract

**Background:**

Mentorship influences career planning, academic productivity, professional satisfaction, and most notably, the pursuit of academic medicine careers. Little is known about the role of mentoring in recruiting Black/African American and Hispanic/Latino residents into academia. The objective of this study was to assess the influence of mentoring on academic medicine career choice among a cohort of racially and ethnically diverse residents.

**Methods:**

A strategic convenience sample of U.S. residents attending national professional conferences between March and July 2010; residents completed a quantitative survey and a subset participated in focus groups.

**Results:**

Of the 250 residents, 183 (73%) completed surveys and 48 participated in focus groups. Thirty-eight percent of residents were white, 31% Black/African American, 17% Asian/other, and 14% Hispanic/Latino. Most respondents (93%) reported that mentorship was important for entering academia, and 70% reported having sufficient mentorship to pursue academic careers. Three themes about mentorship emerged from focus groups: (1) qualities of successful mentorship models; (2) perceived benefits of mentorship; and (3) the value of racial/ethnic and gender concordance. Residents preferred mentors they selected rather than ones assigned to them, and expressed concern about faculty using checklists. Black/African American, Hispanic/Latino, and female residents described actively seeking out mentors of the same race/ethnicity and gender, but expressed difficulty finding such mentors. Lack of racial/ethnic concordance was perceived as an obstacle for minority mentees, requiring explanation of the context and nuances of their perspectives and situations to non-minority mentors.

**Conclusions:**

The majority of residents in this study reported having access to mentors. However, data show that the lack of diverse faculty mentors may impede diverse residents’ satisfaction and benefit from mentorship relationships compared to white residents. These findings are important for residency programs striving to enhance resident mentorship and for institutions working to diversify their faculty and staff to achieve institutional excellence.

## Background

Changing U.S. demographics coupled with increased access to health care through the Affordable Care Act make diversity and inclusion an imperative in academic medicine [1-3]. U.S census data show that the fastest growing populations are racial and ethnic minorities [1]. However, the diversity of U.S. medical school faculty has not changed significantly over the past 20 years [4-6]. Data from the Association of American Medical Colleges (AAMC) indicate that while the total number of faculty increased between 2002 and 2011, the percentage of faculty reporting as Black/African American, American Indian/Alaska Native, and Hispanic/Latino remained the same at 7% [4,6,7]. Exploring multiple strategies to strengthen recruitment and retention, including mentoring and pipeline initiatives, may be critical to advancing diversity in academic medicine.

Mentorship is well documented as influencing faculty advancement, academic productivity, and professional satisfaction [8-11]. During residency, mentoring is viewed as beneficial for career planning, specialty selection, and most notably, the pursuit of academic careers [8,12-16]. However, prior studies indicate that a large proportion of residents lack mentors [13,15]. It is unclear whether racial and ethnic minority residents have mentors or benefit from these relationships in the same manner as their non-minority counterparts. Personal factors, relational challenges, and structural/institutional barriers may compromise mentoring relationships for diverse residents [9,17]. As more residencies adopt structured mentoring programs, an appreciation of racial and ethnic minority residents’ perceptions of mentorship becomes important to ensuring that mentoring experiences are of comparable benefit for diverse groups [8].

Understanding racial and ethnic minority residents’ perceptions of mentorship and its influence on pursuing a career in academic medicine will fill an important research gap and can help inform interventions that foster a more diverse academic workforce. Therefore, we employed a mixed methods approach to: a) assess interest in academic medicine, b) identify factors shaping residents’ perceptions of mentorship, c) ascertain challenges with mentorship, and d) describe the role of mentoring on the pursuit of academic medicine careers.

## Methods

### Study design

This study was developed by the Building the Next Generation of Academic Physicians Initiative, created by the Hispanic Center of Excellence at the Albert Einstein College of Medicine and the Diversity Policy and Programs unit of the AAMC to enhance academic medicine workforce diversity, in collaboration with national medical organizations and academic health centers [18]. Using a triangulation mixed methods design, we combined a quantitative survey with focus groups. Surveys gathered data on career choice, career development, and demographics. Focus groups were conducted with a subset of residents to facilitate an in-depth exploration of mentorship and academic medicine careers. The study was approved by the Institutional Review Boards of the American Institute of Research and Montefiore Medical Center.

### Study sample and recruitment strategy

Using strategic convenience nonprobability sampling, we recruited residents interested in academic careers at the 2010 annual medical conferences of the American Medical Association (AMA), the National Medical Association (NMA), and the National Hispanic Medical Association (NHMA). This strategy allowed for the recruitment of higher proportions of Black and Latino residents. Moreover, it facilitated the collection of data in settings focused on personal and professional development, and perceptually “safer” environments. This latter point is particularly important considering some reports of institutional climates not valuing or supporting racial and ethnic minorities [19-23].

Conference attendees were invited to participate in the study through dissemination of study materials (survey, consent form, focus group recruitment letter) in conference registration bags and by conference announcements. Survey participants were consented, given written instructions, and completed the survey independently. All focus group participants completed the survey prior to the start of focus group discussions. Study participants had the opportunity to enter a raffle for a $250 Apple gift certificate.

### Data collection and measures

Survey and focus group questions were developed based on a literature review of mentorship and academic careers research, the AAMC Graduation Questionnaire, and discussions with experts in diversity workforce research, resident education, and mentorship. The survey and focus group protocols were pilot tested with 30 diverse residents. Changes in question phrasing and sequencing were made based on pilot data and participant feedback.

The final quantitative survey consisted of 23 items, covering 3 domains: career choice, career development, and demographics. For the purposes of this paper, we focused on items describing residents’ interest in academia and perceptions of mentorship. Participants were asked their level of interest in an academic medicine career using the 5-point Likert scale: 1 (very interested), 2 (interested), 3 (neutral), 4 (disinterested), and 5 (very disinterested). Perceptions of mentorship were assessed with the following statements: “I do not have sufficient mentorship to pursue a career in academic medicine” and “I do not know how to utilize mentors to advance my career”, using the 5-point Likert scale (1 = strongly agree, 2 = agree, 3 = neutral, 4 = disagree, 5 = strongly disagree). Lastly, participants were asked to rate the influence of mentors/role models on pursing an academic medicine career utilizing the 5-point Likert scale: 1 (very positive influence), 2 (positive influence), (3 (unsure), 4 (negative influence), and 5 (very negative influence). Demographic variables collected were age, gender, race/ethnicity, specialty, post-graduate year (PGY), and estimated total medical education debt.

Focus groups elicited participants’ perspectives regarding factors shaping the choice of pursuing a career in academic medicine. Each focus group included 7-12 residents and lasted 45-55 minutes, consistent with established qualitative focus group methodology [24]. Trained moderators, who were familiar with the study goals and skilled in focus group techniques, facilitated all focus groups. Participants were asked to describe their understanding of academic medicine, interests in pursing academic careers, timing and factors shaping the decision making process, barriers and facilitators to entering academia, and suggestions for increasing diversity. Focus groups were audio-recorded and transcribed. De-identified transcripts and field notes were checked for accuracy and entered into NVivo 9, a software package for qualitative data analysis (QRS International, Cambridge, MA).

### Analysis

Descriptive analyses were conducted. Response to survey statements were compared across age, gender, race/ethnicity, specialty, PGY, and estimated medical education debt using the non-parametric Kruskal-Wallis test, which allows for non-normal distribution of scores, with significance levels at p ≤ 0.05. Associations between interest in academic medicine careers and attitudes about mentorship were determined using Pearson correlation coefficients. IBM SPSS 19.0 software was used for all quantitative analyses.

Focus group data were analyzed for themes and patterns using a grounded theory approach, a methodology that involves iterative development of theories about what is occurring in the data as they are collected [25,26]. The process develops themes and sub-themes that emerge “from the ground” based on responses to the questions. A multidisciplinary team of five investigators were involved in coding and analyzing the transcripts. Discrepancies in coding were resolved by group consensus. Identified themes and sub-themes were compared across participant groups to assess for differences by race/ethnicity.

## Results

A total of 183 residents returned completed surveys for an overall response rate of 73% (183/250). The sample was 52% female, with 38% self-reporting as White, 31% as Black/African American, 17% as Asian or other, and 14% as Hispanic/Latino. Residents trained in both primary care (43%) and non-primary care (51%) specialties; the majority (58%) were PGY-3 and above. Nearly half (48%) of respondents estimated their medical education debt at more than $150,000.

Forty-eight residents participated in focus groups; 2 focus groups with 7 and 11 self-identified Black/African American residents occurred at the NMA conference, 2 focus groups with 11 and 12 self-identified White or Asian residents occurred at AMA, and one focus group of 7 self-identified Hispanic/Latino residents occurred at NHMA. Demographics were similar to survey respondents, except focus groups included more males (50%), Black/African Americans (37%), Hispanic/Latino (15%), and trainees PGY-3 and above (66%) (Table 1).

**Table 1:** Demographic and specialty characteristics of resident survey respondents and focus group participants

**Characteristic**	**Survey respondents (n = 183) no. (%)**	**Focus group participants (n = 48) no. (%)**
Age (years)		
18-34	156 (85)	38 (80)
≥ 35	17 (9)	5 (10)
Missing	10 (6)	5 (10)
Gender		
Female	95 (52)	22 (46)
Male	83 (45)	24 (50)
Missing	5 (3)	2 (4)
Race/Ethnicity		
Black/African American	57 (31)	18 (37)
Hispanic/Latino	25 (14)	7 (15)
White	69 (38)	16 (33)
Asian/Other	32 (17)	7 (15)
Specialty		
Primary care	79 (43)	21 (44)
Non-primary care	93 (51)	25 (52)
Missing	11 (6)	2 (4)
Post-Graduate Year (PGY)		
PGY-1	22 (18)	9 (19)
PGY-2	28 (21)	7 (15)
PGY-3	55 (30)	15 (31)
PGY-4 and above	51 (28)	17 (35)
Missing	6 (3)	0 (0)
Estimated Medical Education Debt		
< $150,000	88 (48)	25 (52)
> $150,000	87 (48)	22 (46)
Missing	8 (4)	1 (2)

### Quantitative data

The majority of survey respondents (88%) were interested or very interested in academic medicine as a career (Mean Likert Score = 1.84, 95% Confidence Interval = 1.73-1.95), with 8% being neutral. Eight-five percent of Black/African American, 91% of Hispanic/Latino, 92% of Asian/other, and 88% of white residents were interested or very interested in academic careers. There were no significant differences by race and ethnicity, estimated medical education debt, or other resident characteristics (all p > 0.05) (Table 2).

**Table 2:** Residents’ interest in academic medicine careers

**Characteristic**	**How interested are you in academic medicine as a career? Mean Likert Score (95% CI)**
Age (years)	
18–34	1.86 (1.74–1.98)
≥ 35	1.89 (1.71–2.05)
Gender	
Female	1.91 (1.75–2.06)
Male	1.78 (1.63–1.93)
Race/Ethnicity	
Black/African American	1.85 (1.65–2.06)
Hispanic/Latino	1.68 (1.40–1.97)
White	1.90 (1.70–2.09)
Asian/Other	1.81 (1.61–2.00)
Specialty	
Primary care	1.76 (1.60–1.92)
Non-primary care	1.86 (1.71–2.01)
Post-Graduate Year (PGY)	
PGY1	1.97 (1.72–2.22)
PGY2	1.84 (1.71–2.23)
PGY3	1.87 (1.67–2.07)
PGY4 and above	1.63 (1.43–1.82)
Estimated Medical Education Debt	
< $150,000	1.77 (1.61–1.93)
> $150,000	1.90 (1.76–2.05)

Note: Likert scale ranged from 1 (very interested) to 5 (very disinterested).

Overall, 70% of residents reported having sufficient mentorship to purse an academic career with 17% reporting insufficient mentorship and 13% neutral. Among Asian/other residents, 26% agreed or strongly agreed that they did not have sufficient mentorship to enter academia, compared to 16% of Hispanic/Latino, 16% of Whites, and 14% of Black/African American residents. Additionally, 72% of residents knew how to utilize mentors to advance their career, with 13% reporting not knowing how to use mentors and 14% neutral. Nineteen percent of Asian/other residents, 16% of Black/African American, 10% of White, and 8% of Hispanic/Latino residents agreed or strongly agreed that they did not know how to use mentors to advance their careers. There were no significant differences by race and ethnicity, estimated medical education debt, or other resident characteristics (all p > 0.05) (Table 3).

**Table 3:** Residents’ Perception of mentorship on academic medicine careers

**Characteristic**	I do not have sufficient mentorship to pursue a career in academic medicine Mean Likert Score (95% CI)*	I do not know how to utilize mentors to advance my career Mean Likert Score (95% CI)*	**Rate the influence of mentors/role models on pursing an academic medicine career Mean Likert Score (95% CI)†**
Age (years)			
18–34	3.77 (3.61–3.93)	3.88 (3.73-4.04)	1.58 (1.47-1.68)
≥ 35	3.65 (3.02–4.28)	3.41 (2.75–4.07)	2.23 (1.57–2.89)
Gender			
Female	3.72 (3.51–3.52)	3.68 (3.46–3.91)	1.54 (1.40–1.67)
Male	3.78 (3.54–4.02)	3.98 (3.79–4.16)	1.74 (1.56–1.92)
Race/Ethnicity			
Black/African American	3.80 (3.50–4.10)	3.82 (3.49–4.14)	1.63 (1.41–1.85)
Hispanic/Latino	3.50 (3.12–3.88)	3.95 (3.53–4.38)	1.53 (1.28–1.77)
White	3.83 (3.57–4.08)	3.81 (3.60–4.01)	1.70 (1.51–1.90)
Asian/Other	3.70 (3.34–4.06)	3.78 (3.45–4.12)	1.55 (1.33–1.77)
Specialty			
Primary care	3.68 (3.45–3.92)	3.79 (3.57–4.02)	1.64 (1.47–1.80)
Non–primary care	3.88 (3.67–4.09)	3.90 (3.70–4.10)	1.63 (1.48–1.78)
Post-Graduate Year (PGY)			
PGY1	3.64 (3.27–4.00)	3.70 (3.32–4.08)	1.52 (1.24–1.80)
PGY2	3.79 (3.46–4.12)	3.79 (3.49–4.09)	1.53 (1.31–1.74)
PGY3	3.75 (3.46–4.03)	3.89 (3.65–4.12)	1.72 (1.49–1.95)
PGY4 and above	3.86 (3.55–4.17)	3.92 (3.62–4.22)	1.68 (1.49–1.88)
Estimated Medical Education Debt			
< $150,000	3.74 (3.51–3.96)	3.91 (3.70–4.12)	1.72 (1.58–1.86)
> $150,000	3.77 (3.55–3.98)	3.74 (3.53–3.95)	1.56 (1.39–1.73)

*Likert scale ranged from 1 (strongly agree) to 5 (strongly disagree).

†Likert scale ranged from 1 (positive influence) to 5 (negative influence).

The majority of residents (93%) believe that mentors are a positive or very positive influence in the decision to pursue a career in academic medicine, with 6% unsure. Among Hispanic/Latino residents, 100% believed that mentors are positive or very positive influences on the decision to purse academic careers, compared to 97% of Asians/others, 91% of Whites, and 89% of Black/African American residents. Because of numerous small cell sizes (all contingency tables have < 5/cell in > 50% of cells) and expected values less than 1, no chi-square analyses were performed (Table 3).

Associations between interest in academic medicine careers and attitudes about mentorship were examined. Interest in academic medicine negatively correlated with the question “I do not have sufficient mentorship to pursue a career in academic medicine” (r = -0.27, p < 0.01). That is, residents who reported having adequate mentorship were more interested in academic medicine as a career than those with inadequate mentorship. Similarly, participants’ interest in academic medicine negatively correlated with the question “I do not know how to utilize mentors to advance my career” (r = -0.26, p < 0.01). Specifically, residents who were able to work with their mentors to advance their careers were more interested in academia than their counterparts. No correlation between interest in academic medicine careers and the question “Rate the influence of mentors/role models on pursing an academic medicine career” was identified (r = -0.12, p = 0.13).

### Qualitative data

Participants described clinical work, teaching, research, administration, and policy work as the core domains of academic medicine. Primary care respondents, regardless of race/ethnicity, frequently described academic careers as clinical precepting and affiliations with universities more than other respondents. Some respondents conceptualized pursing academic interests through other venues such as local professional societies and opportunities to influence municipal, state, and national healthcare agendas.

Three major themes about mentorship emerged from the focus group data: (1) qualities of successful mentorship models; (2) perceived benefits of mentorship; and (3) the value of racial/ethnic and gender concordance (Additional file 1: Table S1). Successful mentoring was characterized as an engaged and personalized process. Residents preferred a more individualized approach for identifying mentors rather than arbitrary assignments, and expressed concerns about faculty using checklists rather than tailoring mentorship to the specific needs of the mentee. Mentors were described as filling concrete knowledge and process gaps, role modeling desired behaviors, and key to gaining access to academia. Some residents described the need for multiple mentors in order to provide guidance in multifold areas of development.

White residents were the only group that raised concerns over some training programs taking an assembly-line approach to the production of academicians. The same respondents described mass production models as antithetical to the individualized mentoring model necessary to support trainees interested in academia.

Networking was an identified benefit of mentorship that included a sense of increased social capital that opened doors, improved the transparency of processes, and created opportunity. Exposure to professional networks provided awareness and access to well developed systems, preventing the need for mentees to make connections and forge pathways independently. The value of networking was more commonly discussed among non-minority residents.

Black/African American, Hispanic/Latino, and female residents described value in identifying mentors with similar demographics and shared sense of history. The ability to see oneself and one’s future potential in a faculty member added to their perceived value as a mentor. Black/African American and Hispanic/Latino participants described the need to identify mentors with an understanding of their personal and professional career trajectories. Gender and racial/ethnic concordance was described as desirable and encouraging. Incompatibility was perceived as an obstacle for minority mentees, requiring explanation of the context and nuances of their situation to non-minority mentors. Female residents raised concerns about the availability of female mentors in traditionally male-dominated fields (e.g. surgery) and in high-level leadership positions across all disciplines.

Respondents described actively seeking out mentors of the same gender and race/ethnicity, but expressed difficulty finding such mentors. Related to this finding, minority residents described a sense of responsibility for addressing the gaps in minority mentorship for future generations of physicians. This desire to give back, as well as a sense of appreciation and respect for the mentorship they received, were both described as primary drivers in their decision to pursue careers in academic medicine.

## Discussion

This study is among the first to examine the influence of mentoring on academic career choice among a cohort of diverse residents. The majority of residents reported having sufficient mentorship to enter academia, with only 17% lacking adequate mentorship. Our findings demonstrate that access to sufficient mentorship and the ability to use mentors for career advancement were significantly associated with residents’ interest in pursing academic careers. Racial and ethnic minority and women respondents unlike their counterparts expressed a desire and perceived value in having access to concordant individuals to serve as mentors.

Residents defined academic medicine as the joint pursuit of clinical, teaching, research, administrative, and policy work. This definition is consistent with prior descriptions of academic medicine, highlighting the tripartite missions of education, research, and patient care [27,28]. Interestingly, primary care residents, independent of race or ethnicity, conceptualized academic careers as clinical precepting and affiliations with universities more than their counterparts. These associations may reflect primary care residents’ exposure to academia during residency training, which relies heavily on clinic precepting and use of community-based physicians [29]. Future studies should examine how medical students’ and residents’ experiences and training influence their definition of academic medicine.

Consistent with earlier research, our residents described successful mentoring as individualized and engaging; but expressed specific concerns about assigned mentoring programs [13,17,30,31]. Prior studies demonstrate that residents who choose their own mentors were more satisfied [13]. Overuse of checklists to track career development and a perceived mass production culture for building academicians were key concerns for participants. Attention to the mentor-mentee relationship, mentor attributes, and the mentoring process are critical for personal and professional success [32].

Most of our residents had sufficient mentorship to enter academia, which may be a consequence of recent national efforts aimed at improving the learning environment, a better understanding of what constitutes a successful mentor-mentee relationship, and greater attention to residents’ professional development by training programs [33-35]. In addition, our findings may be unique to this subset of residents considering that they were recruited at conferences focused on professional development and networking. Each medical organization provides various leadership activities for residents that connect them with senior physicians and other resources to support their career development. Participation in these and similar medical organizations may be an important factor to consider in examining support networks for residents.

However, some residents revealed that they lacked sufficient mentorship to enter academic medicine. Cain and colleagues similarly report that mentorship was inadequate for obstetric and gynecology residents, particularly for Black/African American, American Indian/Alaska Native, and Hispanic/Latino residents [14]. This may reflect an inability of residents to identify mentors, resident apathy towards mentoring, limited faculty or resident time, lack of mentor skills, and/or incompatible mentor-mentee relationship [17]. Further studies need to clarify why academic medicine mentorship may be insufficient and evaluate interventions to improve mentorship particularly for diverse residents.

We did not identify any significant differences by race and ethnicity to the questions: “I do not have sufficient mentorship to pursue a career in academic medicine” and “I do not know how to utilize mentors to advance my career.” However, racial and ethnic minority residents identified personal factors, relational difficulties, and structural/institutional barriers to achieving successful mentoring relationships [9,17]. In this study, Black/African American and Hispanic/Latino residents noted the shortage of racial and ethnic minority faculty and its implications for finding compatible mentors. Similarly, female residents reported challenges finding female mentors in traditionally male-dominated fields. The lack of fit between mentor and mentee may lead to dissatisfaction; as well as, hinder mentee progress, compromise trust, and illicit feelings of vulnerability or isolation in the mentee [17,36]. Residencies, especially those with structured mentoring programs, should be aware of the value and relevance of congruence and develop methods to facilitate compatibility. Additionally, programs should foster other important qualities of a successful mentoring relationship, namely mutual respect, commitment, effective communication, clear expectations, personal connection, and shared values [37].

As institutions strive to diversify their academic workforce and support their faculty mentors, it is important for residents to be proactive and strategic in securing mentorship. One possibility is for residents to search for mentors outside of their home department or institution. Medical professional organizations (e.g. NMA, NHMA) and professional development seminars may provide opportunities to identify new mentors. Additionally, residency programs that lack racial and ethnic faculty diversity should consider engaging faculty in other departments, from institutional leadership, or from the National Center on Minority Health and Health Disparities Centers of Excellence. An added benefit of this approach is that residents will gain exposure and network with faculty from outside their departments, which may heighten their visibility and enhance opportunities to serve in leadership roles. A potential limitation is the willingness and ability of one department to compensate the efforts (monetarily and with protected time) of faculty members from another department. Creative solutions and additional resources are needed to increase interdepartmental and intra-institutional cooperation for mentoring programs.

This study has several limitations. Our sample included only residents participating in national professional conferences, who may differ from other residents. They may have better professional networks, and greater interest in academic medicine. Therefore, our results may not generalize to all residents. Yet, given our cohorts high interest in academia, they may represent an important group to focus on and cultivate to ensure their professional growth in academic medicine. An additional limitation is that survey and focus group participation was voluntary; data collected may be unduly influenced by participants with strong opinions about mentorship or academic medicine. This study was unable to recruit individuals who identified as American Indian, Alaska Native and Pacific Islanders, and did not distinguish between U.S. medical graduates, U.S.-born international medical graduates, and foreign-born international medical graduates. Additional efforts are needed to assess and document how these residents perceive mentoring and the pursuit of academic medicine careers.

## Conclusions

Mentorship is viewed as important for pursuing a career in academic medicine [10,11,18,38,39]. The majority of residents (70%) reported having sufficient mentorship to enter academia. However, some respondents (17%) reported insufficient mentorship to enter academia or were neutral (13%). Black/African American, Hispanic/Latino, and female residents expressed difficulty in finding mentors of the same race/ethnicity or gender, which was perceived as an obstacle. Our findings indicate that institutional efforts to promote mentorship will not guarantee satisfaction without an appreciation of resident preferences and perspectives. These data support a tailored approach to the development of mentorship programs. Future work to evaluate existing mentorship programs and develop new initiatives to meet the needs of racial and ethnic minority residents are essential for increasing diversity across all faculty ranks in academic medicine.

## Abbreviations

AAMC: Association of American Medical Colleges; AMA: American Medical Association; NHMA: National Hispanic Medical Association; NMA: National Medical Association; PGY: Post-graduate year.

## Competing interests

The authors declare that they have no competing interests.

## Authors’ contributions

BRY participated in acquisition of data, analysis and interperation of data, drafting of the manuscript, critical revision of the manuscript for important intellectual content, administrative support, and study supervision. PFC and SCP participated in analysis and interperation of data, statistical analysis, and critical revision of the manuscript for important intellectual content. NW, SDS, and CEG participated in analysis and interperation of data and critical revision of the manuscript for important intellectual content. NIH participated in analysis and interpretation of data, drafting of the manuscript, and critical revision of the manuscript for important intellectual content. JPS participated in study design, acquisition of data, analysis and interperation of data, drafting of the manuscript, critical revision of the manuscript for important intellectual content, obtaining funding, administrative support, and study supervision. All authors read and approved the final manuscript.

## Pre-publication history

The pre-publication history for this paper can be accessed here:

http://www.biomedcentral.com/1472-6920/14/26/prepub

## Supplementary Material

Additional file 1: Table S1 Themes, Sub-Themes, and Quotes from Residents Participating in Focus Groups. (Expanded). Click here for file
