# Supplementary material for: Mentorship and pursuit of academic medicine careers: a mixed methods study of residents from diverse backgrounds
Source: BMC Med Educ. 2014 Feb 9;14:26. doi: 10.1186/1472-6920-14-26 (PMC3922304; doi:10.1186/1472-6920-14-26)
Supplement: Additional file 1: Table S1 — Themes, Sub-Themes, and Quotes from Residents Participating in Focus Groups. (Expanded). [file 1472-6920-14-26-S1.docx]

Additional file 1: Table S1. Themes, Sub-Themes, and Quotes from Residents Participating in Focus Groups. (Expanded)

| **Theme** | | **Quote** |
| --- | --- | --- |
| Defining Academia | | *“I think academic medicine, it's kind about a catchall for a lot of different things … developing the physicians we have today and also the ones that we're going to be in tomorrow and we're [refining] the field of medicine and discussing where are some current issues that we have, what can we improve upon, what are the things that people keep stumbling over …”* **– Male Resident, White/Asian** |
| **Theme** | **Sub-Themes** | **Quotes** |
| Qualities of Successful Mentorship Models | Engaged and Individualized Process | *“Every so often, like every year or so, [my mentor] will be like, where is your resume, you know, the center of discussion, where’s your 5-year plan, where’s your 10-year plan? How do you plan to make that happen and even though I’m sweating when I’m in there, by the time I leave I feel better about what I’m doing, you know.”* **– Female Resident, White/Asian**  *“… when I went to my residency, I kind of experienced a completely different sort of environment. One which was very nurturing, which was encouraging and one where I saw them not try to force research on everybody. They basically identify the people that showed an interest and love in a certain topic and then they would start putting more resources there and I think that sort of model works much better. For me, that was actually my turning point.”* **– Male Resident, White/Asian**  “It’s just that the way I felt I was going along, she was just always there and very supportive and would give me little bits of advice here and there, and I still keep in contact with her and would still talk a lot about varied things. But she was huge as well as another -- not as much of a mentor but faculty adviser who was very important in my push into the fellowship I want to go into.” **– Female Resident, Black/African American** |
|  | Filling Gaps and Role Modeling | “…it was having a mentor who actually let me shadow him…Because otherwise, it’s really tough to get a feel for having possibly do all that’s required or everything that we think we want to do in any given day but he let me see that it is actually possible with a lot of planning and calendars.” **– Female Resident, Black/African American**  “…having somebody to kind of tell me what it was like in reality without hiding anything, really helped me realize that that is what I wanted to do [academic medicine] despite the barriers...” ***–* Female Resident, Black/African American**  “One of the mentors early on in my career told me that academic medicine is truly the place where you could really advocate for the patient population in all aspects, not only in clinical care but also new discoveries, working with other colleagues, expanding the field, and educating future physicians and really sort of setting the tone of how patient care will be delivered. And so I think for me, my interest in academic medicine is all of that and also, I mean, having heard something like that from a perspective of a mentor and having an adviser going out…” **– Male Resident, White/Asian**  “I still recall some of the great mentors that I had specific clinical approach that I learned that I’ll carry with me I hope to instill that in other people now. And I think that was a big piece for me personally as I got involved in academics.” **– Male Resident, White/Asian**  “I have mentors that are just for personal reasons, mentors for research reasons, mentors for clinical reasons, like it’s very good to break it down because each one -- I mean each one have their strengths and they may have their weaknesses also so it’s always good to continue networking for mentors also.” **– Female Resident, Black/African American** |
|  | Need for Meaningful Engagement | “I think it takes a certain kind of mentor. It's not someone that's assigned to you because now a lot of programs have resident mentorship things and you're supposed to meet with this person, and they’re like have a little checklist, you do this research project, did you do this professionalism, blah, blah, blah. And if you don't hear from me, that's cool, you're doing fine. I think it takes a little bit more than a checklist.” **– Female Resident, White/Asian**  *“I think it's very important that you have very organic sort of relationship, one where there's encouragement and one where there's actual interaction that is meaningful. I think a lot of times - I think another speaker mentioned this - is that these institutions are trying to be factories that just churn out academicians. And I think that's a totally contrived sort of concept that doesn't really work. Where I went to medical school, I feel like that was one of those choices where they basically would say if you don't end up being an academic physician, you're wasting your life; you're a failure. That was kind of the theme that was pushed and that actually turned me off quite a bit.* **– Male Resident, White/Asian**  *“I don't think you can have a factory that churns out academicians and I think that sometimes the mentorship elements are not as emphasized, I think, with medicine becoming kind of more of an engine, kind of bureaucratic engine.”* ***–* Male Resident, White/Asian** |
| Benefits of Mentorships | Networking | *“I think the networking is the biggest thing, like building a big map or as soon as possible. You know, I have people who like already are saying, hey, you’re going to XYZ please, stay. Such and such are doing research there, give them a call. We called them for you already. I’m like, how am I going to fit that into my year, but just the fact that, you know, that network, they’re going to be looking out for you.”* **– Female Resident, White/Asian**  “[I] becomes really evident when you're in there, how do I do this? But I think one of the ways again getting back to it is that I've been able to deal with it is by actually forming strong connections to people both within the department and outside the department. I think the mentor-thing is absolutely correct. …[T]here's just no way you're going to know how to deal with these problems I think by yourself because you'll be reinventing the wheel in so many different ways. I always have people that I go to for specific advice.” **– Male Resident, White/Asian** |
|  | Help Define Path | “I think you see it is easier to kind of see where you expect to be if you have people who’ve already walked that path who you can talk to about it and you asked them like, how do you get here? What are the things that you did? Why did you obtain this? I mean I do not think that –- you know, I think that having that type of mentorship is difficult. I think if you have it is a great thing.” **– Male Resident, Black/African American**  “He talked to me about the future, where shall I apply, what programs were good, what programs weren’t, and why -- he totally tried to -- he also wanted me to do what he does, pulmonary critical care, which I probably won’t be doing. But I think all of those, all of those men were good mentors, showed me what academic medicine was and how he was involved in it and why he likes it.” **– Male Resident, Hispanic/Latino**  “I think mentors in the true sense of the word are one of the things that can help us get to where we need to be and it's something that we can return once we get there.” **– Female Resident, White/Asian**  “[E]very so often like every year or so, she’ll be like, where is your resume, you know, the center of discussion, where’s your 5-year plan, where’s your 10-year plan? How do you plan to make that happen and even though I’m sweating when I’m in there, by the time I leave I feel better about what I’m doing, you know.” **– Female Resident, White/Asian**  I think during my third year of medical school when I first, you know, when you’re first exposed to the wards, interacting with different attendings. … I didn’t know that you could be in the hospital, be in the clinic, be in the lab, interact with residents and interns and medical student to the extent that you can. …[W]hen I started to interact with many different attendings and saw all of the different hats that they wore, I think that’s what made me really interested in pursuing the academic medicine.” **– Female Resident, Black/African American**  “I have several mentors, some women, some not, some minority, others not. And I think I'm in a place where it's almost expected, the kind of academic role but there are people that you see that have been successful and balance their lives and think -- I was just saying the importance of mentorship I thought it was critically important.” **– Female Resident, Black/African American** |
| Concordance with Mentors | Valuing a Shared Sense of History | *“He was probably the biggest Latino doc I know in medicine. […] I was like, “Whoa.” You don’t see that ever. I think he is the only chair, like, Latino chair of medicine that I know, and then he was so just down to earth. […] It was the first time I ever really had a real mentor and a doctor who kind of liked me and talked to me and I’m like, “Okay, cool. That person is totally cool. He is my mentor.”* ***–* Male Resident, Hispanic/Latino**  “I think mentors that I think are the same gender obviously, they’re minority mentors that you could have help and a lot of my mentors are men; they're other ethnicities. But I think I’m in a place where academic medicine is kind of expected of the folks who are coming through the program but when you actually see people who have been successful, it seems like more of a possibility for you.” **– Female Resident, Black/African American**  “Sophomore year, summer of sophomore year college through the minority program… I saw people like me who came from backgrounds like me, actually a person, I should say, which gave me a lot of focus to actually gain an admission and acceptance and matriculation to complete my time in medical school.” **– Female Resident, Hispanic/Latino** |
|  | The Ability to See One’s Future Potential | “In my residency program, there’s a particular track and the director of that track works in academic medicine and works in community medicine, which is what I want to do. So having the opportunity to be with her and work with her on some of the projects that she was doing influenced me to be more interested in academic medicine.” **– Female Resident, Hispanic/Latino**  “[H]aving mentors and realizing that I wanted to do the types of things that they did. I liked the types of opportunities they had and that it was possible for me to do so.” **– Female Resident, Hispanic/Latino**  “I think you see it is easier to kind of see where you expect to be if you have people who’ve already walked that path who you can talk to about it and you asked them like, how do you get here?… it is totally different if you don’t see a path of how to get to where you would like to be.” **– Male Resident, Black/African American** |
|  | Desire to Give Back | “I would like to be a part of maybe a, as you all kind of said, like a university program trying to have the chance to interact with new people, new minds, try to prepare them for his future, and also try to put like all these ideas that we’re bringing here today, trying to create a new perspective of what is going on with people basically, Hispanics in this country.” **– Male Resident, Hispanic/Latino**  “[T]he few people who were welcoming to learn more about my culture and how my struggle through acculturation and the generational gap between my Mom and I has played a role in my kind of growth. Those individuals who have been welcoming to that have also pushed me to kind of continue to on to educate other residents about the struggle that I was maybe going through with the patients in particular.” **– Female Resident, Hispanic/Latino**  “When you were saying like you do not know because you do not have any mentors, I think that kind of underscores the importance of minorities going into academic medicine because you can be that person to influence other individuals. The same absence that you called, you can be that presence for someone.” **– Female Resident, Black/African American**  “Particularly as a minority, I think it’s important that we ask questions that are important to our population that other faculty members may not ask. […] And I think the teaching component is a huge component of that.” **– Female Resident, Black/African American**  “I guess when I figured out during my residency that I have the ability to teach others and make differences and make changes in terms of our board review and our classes. I think that’s what sparked it and to keep me going forward, so during residency.” **– Female Resident, Black/African American**  “[B]ecause of my interest in just being able to contribute to that field, that’s one of the reasons but also mentorship is another, because I feel like I received so much from mentors as undergrad when I was in medical school and they played such a big role in helping me becoming who I am, so I want to pay back.” **– Female Resident, Black/African American** |
|  | Gender and Racial/Ethnic Concordance | “I think having the opportunity to have students see someone in a position of leadership and instruction that might look like them or not look like them, I think that also has an influence on kind of broadening people’s perspective of what medicine is, so I want to be able to be a representative in that regard.” **– Male Resident, Black/African American**  “And I also have a great respect for all of the mentors and attendings that I worked with who has had such a passion for medicine and also had passion for education, and I think that just seeing them made me want to emulate on how they practice and approach medicine so that’s what made me interested in it.” **– Female Resident, Black/African American**  “I think the obvious challenge is that the people that we would consider to be our mentors to help us advance in this process don’t look like us, and in order to understand that and still get the most benefit out of the relationship, we’re not going to have to think, I am a minority trying to enter a field where there’s not a lot of minorities. We’re going to have to say, I’m a minority entering a field where there a lot of people that can help me that may not understand my situation, so it’s up to me to explain my situation and get the type of feedback that would be productive to my advancement. And I think that’s a role that we don’t ask a lot of people to do but being minority, we're always in that role, but it depends on your mindset and how earnestly you work towards achieving your success.” **– Female Resident, Black/African American**  “[I]f you don't have that constant mentor, I think it makes it a lot more difficult. And I think particularly for women, women in surgery, there aren't women. There are very few female full professors in any department. So you're like, hmm, it's hard to get there, why bother. And a lot of my female colleagues have said that.” **– Female Resident, White/Asian** |
